# Fast dictionary learning from incomplete data

**DOI:** 10.1186/s13634-018-0533-0

**Published:** 2018-02-22

**Authors:** Valeriya Naumova, Karin Schnass

**Affiliations:** 1 Simula Metropolitan Center for Digital Engineering, Martin Linges 25, Fornebu, 1325 Norway; 2 Department of Mathematics, University of Innsbruck, Technikerstraße 13, Innsbruck, 6020 Austria

**Keywords:** Dictionary learning, Sparse coding, Sparse component analysis, Thresholding, K-means, Erasures, Masked data, Corrupted data, Inpainting

## Abstract

This paper extends the recently proposed and theoretically justified iterative thresholding and *K* residual means (ITKrM) algorithm to learning dictionaries from incomplete/masked training data (ITKrMM). It further adapts the algorithm to the presence of a low-rank component in the data and provides a strategy for recovering this low-rank component again from incomplete data. Several synthetic experiments show the advantages of incorporating information about the corruption into the algorithm. Further experiments on image data confirm the importance of considering a low-rank component in the data and show that the algorithm compares favourably to its closest dictionary learning counterparts, wKSVD and BPFA, either in terms of computational complexity or in terms of consistency between the dictionaries learned from corrupted and uncorrupted data. To further confirm the appropriateness of the learned dictionaries, we explore an application to sparsity-based image inpainting. There the ITKrMM dictionaries show a similar performance to other learned dictionaries like wKSVD and BPFA and a superior performance to other algorithms based on pre-defined/analytic dictionaries.

## Introduction

Many notable advances in modern signal processing are based on the fact that even high-dimensional data follows a low complexity model. One such model, which has become an important prior for many signal processing tasks ranging from denoising and compressed sensing to super resolution, inpainting and classification, is sparsity in a dictionary [1–8]. In the sparse model, each datum (signal) can be approximated by the linear combination of a small (sparse) number of elementary signals, called atoms, from a pre-specified basis or frame, called dictionary. In mathematical terms, if we represent each signal by a vector $y_{n} \in \mathbb {R}^{d}$ and collect the entire dataset in the matrix $Y = (y_{1},\ldots, y_{N}) \in {\mathbb {R}}^{d \times N},$ the sparse model can be formalised as

1 $$  Y = \Phi X\ \text{and}\ X\ \text{is sparse.}  $$

Here, the dictionary matrix *Φ* contains *K* normalised vectors (atoms) *ϕ*_*k*_, stored as columns in $\Phi = (\phi _{1}, \ldots, \phi _{K}) \in {\mathbb {R}}^{d \times K},$ and each vector column $x_{n} \in \mathbb {\mathbb {R}}^{K}$ of the matrix $X = (x_{1},\ldots, x_{N}) \in {\mathbb {R}}^{K \times N}$ contains only few non-zero entries. Since the model expressed in Eq. (1) has proven to be very useful in signal processing, the natural next question is how to automatically learn a dictionary *Φ*, providing sparse representations for a given data class. This problem is also known as dictionary learning, sparse coding or sparse component analysis. By now, there exist not only a multitude of dictionary learning algorithms to choose from [9–16] but also theoretical results have started to accumulate [17–26]. As our reference list is necessarily incomplete, we also point to the surveys [8, 27] as trailheads for algorithms and theory respectively.

**Fig. 1:**
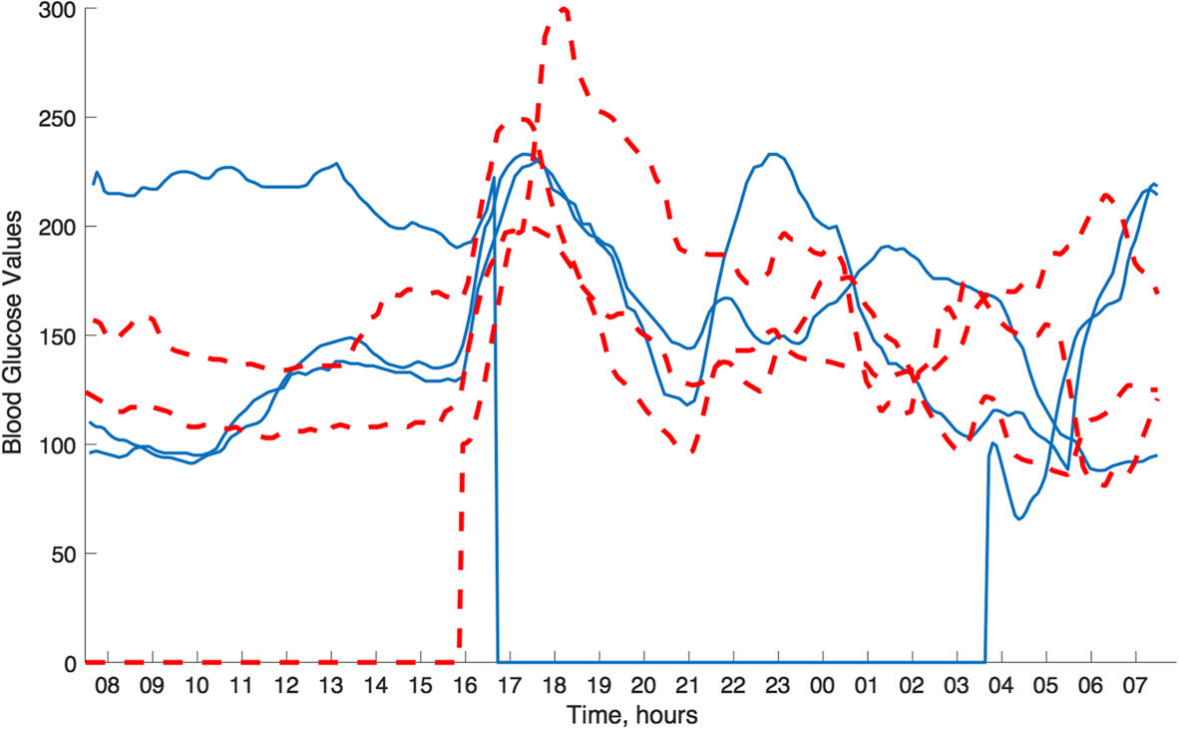
Examples of blood glucose profile of two patients (solid and dashed lines, respectively). Each curve represents a blood glucose profile for a 24-h period from 08:00 till 07:59 the next day during a 3-day inpatient stay. Notice signal dropouts of several hours for two out of six glucose traces

One common assumption on which all algorithms and associated theories are based is that large numbers of clean signals are available for learning the dictionary. However, this assumption might not be valid in actual applications. Therefore, in this paper, we consider the following problem: How do we learn a dictionary when there are only a few or no clean training signals available? This problem naturally arises in various application domains from environmental surveillance, health care to automotive manufacturing, where the data of interest are measured by sensors. As signals from sensors can often be incomplete or contain erroneous measurements due to sensor dropouts or need for recalibration respectively, the amount of clean and reliable data for performing predictive tasks becomes a real issue. As an illustrative example, in Fig. 1, we provide examples of blood glucose traces from two patients as measured by a commercially available continuous glucose monitoring sensor. One can observe that, despite mandatory calibration procedures of the device several times a day, the device quite often returns obviously wrong, e.g. rapidly oscillating, estimations of the blood glucose level and suffers from frequent signal dropouts [28].

To solve the problem of learning from incomplete data, we propose an algorithm called *Iterative Thresholding and K residual Means for Masked data* (ITKrMM). As the name suggests, it is built upon the inclusion of a signal corruption model into the theoretically-justified and numerically efficient *Iterative Thresholding and K residual Means* (ITKrM) algorithm [29].

In order to model the data corruption/loss process, we adapt the concept of the binary erasure channel. In this model, the measurement device sends a value and the receiver either receives the value or receives a message that the value was not received (‘erased’). The model is used frequently in information theory due to its simplicity and its abstraction towards modelling various types of data losses. At the same time, this setting provides information on the location of the erasures and, thus, we can employ the concept of a mask *M* to describe the corrupted data as *My*. Without loss of generality, we will think of a mask *M* as orthogonal projection onto the linear span of vectors from the standard basis (*e*_*j*_)_*j*_ or simply as diagonal matrix with *M*(*j*,*j*)∈{0,1}. We further extend the algorithm to account for the presence of a low-rank component in the data. Such components appear in many real-life signals and, as we will illustrate below, should be treated cautiously in the considered context.

To evaluate the accuracy and efficiency of the algorithm, we perform various numerical tests on synthetic and image data. We also confirm the appropriateness of the learned dictionaries by successfully using them for an image inpainting task.

The dictionary learning community does not directly address the problem under consideration. However, dictionaries learned or refined from corrupted data appear in the image processing community, where they, among other tasks, are used for inpainting. Examples include weighted KSVD (wKSVD) [30, 31], an adaption of the KSVD algorithm to handling non-homogenous noise in signals as well as missing values, and the Beta-Bernoulli Process Factor Analysis (BPFA) [32], a parameter free Bayesian algorithm, that learns dictionaries for inpainting also from corrupted data. As we will see, the main advantage of ITKrMM over the wKSVD algorithm is a significant reduction of computational cost, from around 3.5 h to 18 min in our experiments on image data, while providing similar approximation power and inpainting results. On the other hand, compared to BPFA, we observe similar computational complexity but a much higher consistency between the dictionaries learned from corrupted and uncorrupted data, which is also reflected in the better approximation power of the dictionary and inpainting performance, especially for middle and low corruption levels.

**Contribution:** This paper provides an efficient and simple algorithm for dictionary learning from incomplete data and the recovery of the low-rank component also from incomplete data. Compared to its closest dictionary learning counterparts, wKSVD and BPFA, it combines the best of both worlds, meaning consistent and performant dictionaries like wKSVD at the computational cost of BPFA.

**Outline:** The paper is organised as follows: Section 2 contains the complete problem setup, explaining the combined low-rank and sparse model and as well as the corruption model. The ITKrMM algorithm for dictionary recovery is introduced in Section 3. An adaptation of this algorithm for recovery of the low-rank component from incomplete data together with a short discussion of related works in the field of matrix completion and dimensionality reduction is provided in Section 4. Section 5.1 contains extensive simulations on synthetic data, while in Section 5.2, we compare the learned dictionaries to those of wKSVD and BPFA for image data and use them for inpainting. Finally, Section 6 offers a snapshot of the main contributions and points out open questions and directions for future work.

**Notation:** Before finally lifting the anchor, we provide a short reminder of the standard notations used in this paper. For a matrix *A*, we denote its (conjugate) transpose by *A* ^⋆^ and its Moore-Penrose pseudo inverse by *A* ^*†*^. By *P*(*A*), we denote the orthogonal projection onto the column span of *A*, i.e. *P*(*A*)=*A* *A* ^*†*^, and by *Q*(*A*), the orthogonal projection onto the orthogonal complement of the column span of *A*, that is $Q(A) = {\mathbb {I}}_{d} - P(A)$, where ${\mathbb {I}}_{d}$ is the identity operator (matrix) in ${\mathbb {R}}^{d}$.

The restriction of the dictionary *Φ* to the atoms indexed by *I* is denoted by *Φ*_*I*_, i.e. $\Phi _{I}=(\phi _{i_{1}},\ldots, \phi _{i_{S}})\phantom {\dot {i}\!}$, *i*_*j*_∈*I*. The maximal absolute inner product between two different atoms is called the coherence *μ* of a dictionary, $\phantom {\dot {i}\!}\mu =\max _{k \neq j}|\left \langle {\phi _{k}},{\phi _{j}}\right \rangle |$, and encapsulates information about the local dictionary geometry.

## Problem setup

Our goal is to learn a dictionary *Φ* from corrupted signals *M*_*n*_ *y*_*n*_, under the assumption that the signals *y*_*n*_ are sparse in the dictionary *Φ*. There are some notable differences in this problem setting compared to the uncorrupted situation. First, we cannot without loss of generality assume that the corrupted signals are normalised, since the action of the mask distorts the signal energy, ∥*M* *y*∥_2_≤∥*y*∥_2_, which makes simple renormalisation impossible.

Another issue in modelling a natural phenomenon is that the signals might not be perfectly sparse but can only be modelled as the orthogonal sum of low-rank and sparse components. An example for such signals are images, where one usually subtracts the foreground or, in other words, the signal mean before learning the dictionary, which consequently will consist of atoms with zero mean [9]. Without taking into account the existence of the low-rank component, one would likely end up with a very ill-conditioned and coherent dictionary, where most atoms are distorted towards the low-rank component.

Similarly, in the example of the blood glucose data (see Fig. 1), we can observe that the signals vary around a baseline signal and that imposing a sparse structure in a dictionary makes sense only after subtracting this common component. As before, the atoms in this dictionary should then be orthogonal to the baseline signal.

In the case of uncorrupted signals, one can simply determine the common low-rank component *Γ*=(*γ*_1_…*γ*_*L*_) using one’s preferred method such as a singular value decomposition and subtract its contribution from the signals via $\tilde {y}_{n}= Q(\Gamma)y_{n}$. Then, in a second separate step, one can run the dictionary learning algorithm on the modified signals $\tilde {y}_{n}$ and the resulting atoms will automatically be orthogonal to the low-rank component *Γ*. However, in the case of corrupted signals, the action of the masks destroys the structure. So, while the dictionary is orthogonal to the low-rank component, *Φ* ^⋆^ *Γ*=0, this orthogonality is not preserved by the action of the mask, that is *Φ* ^⋆^ *M* *Γ*≠0. As we will see later, the consequence of this effect is that we have to take the presence of the low-rank component into account when learning the sparsifying dictionary. Moreover, before even going to the dictionary learning phase, we have to find a strategy to recover the low-rank component from the corrupted signals.

The third difference is that we get additional constraints on the dictionaries in order for them to be recoverable. In the case of uncorrupted signals, the main criterion for a dictionary to be recoverable is that its coherence scales well with the average sparsity level *S* of the signals (*S* *μ* ^2^≲1, [29]) and that all atoms are somewhat equally and independently used. In our scenario, where we want to learn a dictionary from corrupted data, we impose another criterion for the recoverability of the dictionary, which is the robustness of the dictionary to corruption. For instance, we will not have a chance to recover an atom *ϕ*_*k*_ if its presence in a signal always triggers the same corruption pattern *M*_0_ which distorts the atom, *M*_0_ *ϕ*_*k*_≠*ϕ*_*k*_. This means that we have to assume some sort of independence between the signals *y*_*n*_ and the corruption, represented by the masks *M*_*n*_. Similarly, it will be very hard to recover a dictionary, whose incoherence is not robust towards corruption. To avoid this complication, we assume that the dictionary and the low-rank component consist of atoms, which are incoherent with the standard basis, that is ∥*ϕ*_*k*_∥_*∞*_≪1 resp. ∥*γ*_*ℓ*_∥_*∞*_≪1. We will also refer to these atoms, where the signal energy is well spread over the coordinates, as flat atoms, as opposed to spiky atoms, where the signal energy is concentrated on one (or a few) coordinate(s), ∥*ϕ*_*k*_∥_*∞*_=1. A more detailed discussion why this is a suitable assumption can be found in Section 3. We point out, however, that this assumption is in line with the potential application of the learned dictionaries to signal reconstruction tasks such as inpainting. There the information in the corrupted part of an image needs to be encoded by the rest of the image, which is the case if the image is sparsely represented by flat atoms.

Incorporating these considerations into the signal model previously used for the analysis of the ITKM algorithms [29], we arrive at the following model, which will be a foundation for the development and justification of the algorithm and for a future theoretical analysis.

### Signal model and assumptions

Given a *d*×*L* low-rank component *Γ* with $\Gamma ^{\star } \Gamma = {\mathbb {I}}_{L}$ and a *d*×*K* dictionary *Φ*, where *Γ* ^⋆^ *Φ*=0 and *L*≪*K*, the signals are generated as

2 $$\begin{array}{*{20}l} y= {s} \cdot \frac{\Gamma {v} + \Phi x +{r}}{\sqrt{1+\|{r} \|_{2}^{2}}} \approx {s} (\Gamma {v} + \Phi_{I} x_{I}), \end{array} $$

where $\|{v}\|_{2}^{2}+ \|x\|^{2}_{2} = 1, |I|=S$, and *r*=(*r*(1)…*r*(*d*)) is a noise vector of a centred subgaussian random vector. The scaling parameter *s* is distributed between *s*_min_ and *s*_max_ and accounts for signals with different energy levels.

The low-rank component is assumed to be present in every (most signals) and irreducible, meaning the coefficients *v* are dense and ${\mathbb {E}}({v}{v}^{\star })$ is a diagonal matrix. Also, the average contribution of a low-rank atom should be larger than that of a sparse atom, ${\mathbb {E}}(|{v}(\ell)|)\gg {\mathbb {E}} (|x(k)|)$. At the same time, the size of the low-rank component is assumed to be much smaller than sparsity level, which in turn is much smaller than the signal dimension, *L*≪*S*≪*d*.

The sparse coefficients *x* should be distributed in a way that for every single signal, only *S* entries in *x* are effectively non-zero. All atoms *ϕ*_*k*_ should be irreducible and on average contribute equally to the signals *y*_*n*_. Specifically, no two atoms should always be used together, since in this case, they could be replaced by any other two atoms with the same span. For a more detailed discussion of admissible coefficient models, we refer to [29].

For those not intimately acquainted with dictionary learning, it might be helpful to keep in mind the following simple model for the subsequent derivations: constant scale and no noise. The low-rank component is one-dimensional, *L*=1, and the low-rank coefficients are equally Bernoulli distributed ±*c*_*v*_. The sparse coefficients are constructed by choosing a support *I* of size *S* uniformly at random and setting *x*(*k*)=±*c*, iid equally Bernoulli distributed, for *k*∈*I* and *x*(*k*)=0 else. In other words, the coefficients restricted to the support are a scaled Rademacher sequence. Following the above considerations concerning the scalings, we have $c_{v}^{2} + S\cdot c^{2} =1$ and *c*_*v*_≫*c* *S*/*K*.

Similar to the signal model, we also discuss our corruption model.

### Corruption model and assumptions

As mentioned above, the corruption of a signal *y* is modelled by applying a mask *M*, where we assume that the distribution of the mask is independent of the signal distribution. By receiving a corrupted signal, we understand that we have access both to the corrupted signal *My* and the location of the corruption in form of the mask *M*, meaning we receive the pair (*M* *y*,*M*).

For the development and later on testing of the algorithms, we will keep two types of corruption in mind. The first type are random erasures, where the *j*th coordinate is received with probability *η*_*j*_ independently of the reception of the other coordinates, meaning *M*(*j*,*j*)∼*B*(*η*_*j*_) are independent Bernoulli variables.

The second type are burst errors or sensor malfunctions. We model them by choosing a burst length *τ* and a burst start *t*, according to a distribution *ν*_*τ*,*t*_. Based on *τ* and *t*, we then set *M*(*j*,*j*) =0 for *t*≤*j*<*t*+*τ* and *M*(*j*,*j*)=1 else. One simple realisation of such a distribution would be to have no burst, *τ*=0, with probability *θ* and a burst of fixed size, *τ*=*T*, which corresponds, for instance, to the time the sensor needs to be reset, with probability 1−*θ*. The burst start could be uniformly distributed, if the sensor is equally likely to malfunction throughout the measurement period or, for instance, with a higher weight on part of the coordinates, if the sensor is more likely to malfunction during part of the measurement period, for instance, during the night.

Having defined our problem setup, we are now ready to address the recovery of the dictionary from corrupted data.

## Dictionary recovery

We will use the iterative thresholding and *K* residual means (ITKrM) algorithm [29], as a base for recovering the dictionary. It belongs to the class of alternating projection algorithms, which alternate between sparsely approximating the signals in the current version of the dictionary and updating the dictionary based on the sparse approximations. As the name suggests, ITKrM uses thresholding as sparse approximation algorithm and residual averages for the dictionary update and as such has the advantage of being computationally light and sequential. Further, there are theoretical results concerning its local convergence and good experimental results concerning its global convergence. Additionally, it is easier to incorporate the information about corruption into a dictionary update scheme that uses averages than into one that uses higher order statistics such as singular vectors. These observations make ITKrM a promising starting point.



To see how we have to modify the algorithm to deal with corrupted data, it will be helpful to understand how ITKrM works. ITKrM can be understood as fixed point iteration, meaning the generating dictionary *Φ* is a fixed point and locally, around the generating dictionary, one iteration of ITKrM is a contraction, $\left \|\phi _{k} - \frac {{\bar \psi }_{k}}{\| {\bar \psi }_{k}\|_{2}}\right \|_{2} < \kappa \|\phi _{k} -\psi _{k}\|_{2}$ for all *k* and some *κ*<1. We refer to [29] for details, but for the sake of completeness, we provide some perhaps intuitive background for both the fixed point and the contraction property.

Assume for a moment that the signals follow the simplest sparse model, that is, they are perfectly *S*-sparse in a generating dictionary *Φ*, meaning $\phantom {\dot {i}\!}y_{n} = \Phi _{I_{n}} x_{n}(I_{n})$ for some |*I*_*n*_|=*S* and *x*_*n*_(*i*)≈±*c* for *i*∈*I*_*n*_, compared to the model presented in Section 2. In particular, they all have the same scaling and contain neither a low-rank component nor are they contaminated by noise. If we are given the generating dictionary as input dictionary, *Ψ*=*Φ*, then as long as the dictionary is not too coherent compared to the sparsity level, *μ* ^2^ *S*≲1, thresholding will recover the generating support, meaning $I_{n}^{t} = I_{n}\phantom {\dot {i}\!}$. Provided that the generating support was always recovered, we have $P\left ({\Psi }_{I_{n}^{t}}\right)y_{n}= P(\Phi _{I_{n}})y_{n} = y_{n}\phantom {\dot {i}\!}$ and before normalisation the updated atom takes the form

$$\begin{array}{*{20}l} {\bar\psi}_{k}&= \sum_{n: k\in I_{n}} P(\phi_{k}) y_{n} \cdot \text{sign}(\left\langle{\phi_{k}},{y_{n}}\right\rangle) \notag \\ &= \sum_{n: k\in I_{n}} |\left\langle{\phi_{k}},{y_{n}}\right\rangle| \cdot \phi_{k}.  \end{array} $$

This means that the output dictionary is again the generating dictionary ${\bar \Psi } =\Phi $ or, in other words, that the generating dictionary is a fixed point of ITKrM. Note also that before normalisation, the updated atom consists of roughly *N*_*k*_=*♯*{*n*:*k*∈*I*_*n*_} scaled copies of itself because |〈*ϕ*_*k*_,*y*_*n*_〉|≈|*x*_*n*_(*k*)|≈*c* and therefore

3 $$\begin{array}{*{20}l}  {\bar\psi}_{k} \approx \sum_{n: k\in I_{n}} c \phi_{k} = c N_{k} \phi_{k}. \end{array} $$

To provide insight why one iteration of ITKrM acts as contraction, assume again that we know all generating supports *I*_*n*_ and that our current estimate for the dictionary consists of all generating atoms except for the first one, *ψ*_*k*_=*ϕ*_*k*_ for *k*≥2. For the first atom, we only have some (poor) approximation, which is, however, still incoherent with all other atoms, 1>|〈*ψ*_1_,*ϕ*_1_〉|≫|〈*ψ*_1_,*ϕ*_*k*_〉|≈*d* ^−1/2^ for *k*≥2, or, in other words, the current estimate *ψ*_1_ contains more of the first than of any other generating atom. As before, one iteration of ITKrM will preserve all atoms *ψ*_*k*_=*ϕ*_*k*_ for *k*≥2 and on top of that contract *ψ*_1_ towards *ϕ*_1_. To see this, observe that as long as the current estimate contains more of the first than of any other generating atoms, |〈*ψ*_1_,*ϕ*_1_〉|≫|〈*ψ*_1_,*ϕ*_*k*_〉|, whenever 1∈*I* for *y*=*Φ*_*I*_ *x*(*I*), we have

$$ P(\psi_{1})y = P(\psi_{1}) \Phi_{I} x(I) \approx x(1) P(\psi_{1}) \phi_{1}.   $$

and, similarly,

$$\begin{array}{@{}rcl@{}} y - P({\Psi}_{I}) y &=& x(1) \left[\phi_{1} - P({\Psi}_{I}) \phi_{1}\right] \\ &\approx& x(1)\left[\phi_{1} - P(\psi_{1}) \phi_{1}\right]. \end{array} $$

Combining the two estimates and noting that sign(〈*ψ*_1_〉*y*_*n*_)=*x*_*n*_(1), we get

$$\begin{array}{*{20}l} {\bar\psi}_{1}&=\sum_{n: 1\in I_{n}} \left[{\mathbb{I}}_{d} - P\left({\Psi}_{I_{n}}\right) + P(\psi_{1})\right] y_{n} \\ &{\kern4cm} \times \text{sign}(\left\langle{\psi_{1}},{y_{n}}\right\rangle) \\ & \approx \sum_{n: 1\in I_{n}} x_{n}(1)\text{sign}(\left\langle{\psi_{1}},{y_{n}}\right\rangle) \cdot \phi_{1}  \\ &\approx \sum_{n: 1\in I_{n}} |x_{n}(1)| \cdot \phi_{1},  \end{array} $$

which shows that also, a poor approximation of ${\bar \psi }_{1}$ is quickly contracted towards the generating atom *ϕ*_1_.

In summary, for our modifications, we have to ensure that both the fixed point and the contraction property are preserved. To start with, we again assume that the corrupted signals have equal scale, contain no low-rank component, and are not contaminated by noise but are perfectly *S*-sparse, that is $\phantom {\dot {i}\!}M_{n} y_{n} = M_{n} \Phi _{I_{n}} x_{n}(I_{n})$. First, observe that a corrupted signal *M*_*n*_ *y*_*n*_ is not sparse in the generating dictionary *Φ* but in its corrupted version *M*_*n*_ *Φ*,

$$\begin{array}{*{20}l} M_{n} y_{n} = M_{n}\Phi_{I_{n}} x_{n}(I_{n}) = \sum_{i\in I_{n}} x_{n}(i) M_{n} \phi_{i}.  \end{array} $$

Still, we can recover the support *I*_*n*_ via thresholding using the corrupted dictionary *M*_*n*_ *Φ* since we have access to the mask *M*_*n*_. However, we have to take into account that, strictly speaking, the corrupted dictionary is not actually a dictionary in the sense that its columns are not normalised. Depending on the shape of the atoms, flat or spiky, and the amount of corruption, $\|M_{n}\|_{F}^{2}$, the norm of the corrupted atoms ∥*M*_*n*_ *ϕ*_*k*_∥_2_ can vary between 0 and 1 corresponding to the extreme cases of being completely destroyed, *M*_*n*_ *ϕ*_*k*_=0, or perfectly preserved, *M*_*n*_ *ϕ*_*k*_=*ϕ*_*k*_. Therefore, the proper dictionary representation of the corrupted signal is

4 $$\begin{array}{*{20}l} M_{n} y_{n} = \sum_{i\in I_{n}:\atop M_{n} \phi_{i} \neq 0} x_{n}(i) \|M_{n} \phi_{i}\|_{2} \cdot \frac{M_{n} \phi_{i}}{\|M_{n} \phi_{i}\|_{2}}, \end{array} $$

and in order to recover the support *I*_*n*_ via thresholding, we have to look at the inner products between the corrupted signal and the renormalised non-vanishing corrupted atoms,

$$\begin{array}{@{}rcl@{}} I_{n}^{t} &=&\arg\max_{I: |I| = S} \sum_{i\in I: \atop M_{n} \phi_{i} \neq 0} \frac{| \left\langle{M_{n} \phi_{i}},{y_{n}}\right\rangle|}{\|M_{n} \phi_{i}\|_{2}}  \\ &=& \arg\max_{I: |I| = S}\sum_{i\in I} \| P(M_{n} \phi_{i}) M_{n} y_{n}\|_{2}.  \end{array} $$

Looking back at the representation of a corrupted signal in the properly scaled corrupted dictionary (4), we can also see why we assume flatness of the dictionary atoms, i.e. ∥*ϕ*_*k*_∥_*∞*_≪1 for all *k*. In the ideal case where for all atoms *ϕ*_*k*_ we have $\|\phi _{k}\|_{\infty }=1/\sqrt {d}$, the energy of the corrupted atoms will be constant $\|M_{n} \phi _{k}\|_{2}=\|M_{n}\|_{F}/\sqrt {d}$ so the dynamic range of the corrupted signal with respect to the corrupted normalised dictionary is the same as the original dynamic range,

$$\begin{array}{*{20}l} \frac{\max_{i \in I_{n}} |x(i)| \cdot \|M_{n} \phi_{i}\|_{2}}{\min_{i \in I_{n}}|x(i)| \cdot \|M_{n} \phi_{i}\|_{2}} = \frac{\max_{i \in I_{n}} |x(i)| }{\min_{i \in I_{n}}|x(i)|}  \end{array} $$

However, the less equally distributed over the coordinates the energy of the undamaged atoms is, the more the energy of the corrupted atoms varies. This leads to an increase of the dynamic range, which in turn makes it harder for thresholding to recover the generating support.

The second reason for assuming flat atoms is the increase in coherence caused by the corruption. If the coherence of two flat atoms is small, this means that their inner product is a sum of many small terms with different signs eventually almost cancelling each other out. Such a sum is quite robust under (random) erasures, since both negative and positive terms are erased. On the other hand, if the energy of two atoms is less uniformly distributed, small coherence might be due to one larger entry in the sum being cancelled out by many small entries. Thus, the erasure of one large entry can cause a large increase in coherence, which again decreases the chances of thresholding recovering the generating support.

Finally, to see that the flatness assumption is not merely necessary due to the imperfection of the thresholding algorithm for sparse recovery, assume that the atoms of the generating dictionary are combinations of two diracs $\phi _{i}=(\delta _{i} - \delta _{(i+1)})/\sqrt {2}$, that the coefficients follow our simple sparse model and that the corruption takes the form of random erasures, i.e. *M*_*n*_(*j*,*j*) are iid Bernoulli variables with *P*(*M*_*n*_(*j*,*j*)=0)=*η*. For large erasure probabilities, *η*>1/2, on average, about half of the maximally 2*S* non-zero entries of the signals will be erased and so the Dirac dictionary *ψ*_*i*_=*δ*_*i*_ or rather its erased version will provide as plausible an *S*-sparse representation to the corrupted signals as the original dictionary *Φ*.

To see how to best modify the atom update rule, we first consider the case, where the corruption occurs always in the same locations, meaning *M*_*n*_=*M*. Since we never observe the atoms on the coordinates where *M*(*k*,*k*)=0, we can only expect to learn the corrupted dictionary *M* *Φ*=(*M* *ϕ*_1_…*M* *ϕ*_*k*_) or rather its normalised version (*M* *ϕ*_*k*_/∥*M* *ϕ*_*k*_∥_2_). On the other hand, the problem reduces to a simple dictionary learning problem for *M* *Φ* instead of *Φ* with update rule,

$$\begin{aligned} M{\bar\psi}_{k} &= \sum_{n: k\in I_{n}^{t}} \left[{\mathbb{I}}_{d} - P\left(M{\Psi}_{I_{n}^{t}}\right) + P(M\psi_{k})\right] M y_{n} \\ &\quad\times \text{sign}(\left\langle{\psi_{k}},{M y_{n}}\right\rangle),  \end{aligned} $$

where we have used the fact that the projection onto a subdictionary is equal to the projection onto its normalised version and that sign(〈*M* *ψ*_*k*_,*M* *y*_*n*_〉/∥*M* *ψ*_*k*_∥_2_)=sign(〈*ψ*_*k*_,*M* *y*_*n*_〉). Provided that thresholding always recovers the correct support *I*_*n*_, we can conclude directly from above that the normalised corrupted dictionary will be a fixed point and that the update rule will contract towards it. Indeed, for any corruption pattern *M*, we know that before normalisation, an updated atom $M{\bar \psi }_{k}$ will be contracted towards *N*_*k*_=*♯*{*n*:*k*∈*I*_*n*_} scaled copies of the corrupted generating atom *M* *ϕ*_*k*_,

$$\begin{aligned} \sum_{n: k\in I_{n}} & \left[{\mathbb{I}}_{d} - P\left(M{\Psi}_{I_{n}}\right) + P(M\psi_{k})\right] M y_{n}\\ &{\kern4cm} \times \text{sign}\left(\left\langle{\psi_{k}},{M y_{n}}\right\rangle\right) \\ & \rightsquigarrow \quad N_{k} \cdot c M\phi_{k} = c \cdot \sum_{n: k\in I_{n}} M \phi_{k}. \end{aligned} $$

This suggests that for the case of different corruption patterns *M*_*n*_, we can simply replace *M* by *M*_*n*_ and the updated atom will be contracted towards the sum of scaled copies of the generating atom, corrupted with the different patterns,

$$\begin{aligned} \sum_{n: k\in I_{n}} &\left[{\mathbb{I}}_{d} - P\left(M_{n}{\Psi}_{I_{n}}\right) + P(M_{n}\psi_{k})\right] M_{n} y_{n} \\ &{\kern4cm}\times \text{sign}(\left\langle{\psi_{k}},{M y_{n}}\right\rangle)\\ &\rightsquigarrow\quad c \cdot \sum_{n: k\in I_{n}} M_{n} \phi_{k}.  \end{aligned} $$

Then, to reconstruct the generating atom from the sum of its corrupted copies, we just need to count how often we observe the atom on each coordinate. If each coordinate has been observed at least once, we can obtain the generating atom simply by rescaling according to the number of observations, meaning we calculate

$$\begin{aligned} {\bar\psi}_{k}&=\sum_{n: k\in I_{n}^{t}} \left[{\mathbb{I}}_{d} - P\left(M_{n}{\Psi}_{I_{n}^{t}}\right) + P(M_{n} \psi_{k})\right] M_{n} y_{n} \\ &{\kern4cm}\times \text{sign}(\left\langle{\psi_{k}},{M_{n} y_{n}}\right\rangle) \\ &\text{and}\quad W_{k}=\sum_{n: k\in I_{n}^{t}} M_{n}, \end{aligned} $$

set $\bar {\bar \psi }_{k} = W_{k}^{\dagger } {\bar \psi }_{k}$ and output ${\bar \Psi }=\left (\frac {\bar {\bar \psi }_{1}}{\|\bar {\bar \psi }_{1}\|_{2}}, \ldots, \frac {\bar {\bar \psi }_{K}}{\|\bar {\bar \psi }_{K}\|_{2}}\right).$The last detail we need to account for is the possible existence of a low-rank component *Γ*; other than noise or different signal scalings, its contribution cannot be expected to average out once we have enough observations. Fortunately, removing the low-rank component is quite straightforward, once we have a good estimate $\tilde {\Gamma }$ with $P(\tilde {\Gamma })\Gamma \approx \Gamma $. If a signal contains a low-rank component, then the corrupted signal will contain the corrupted component, *M* *y*=*M* *Γ* *v*+*M* *Φ*_*I*_ *x*(*I*), and we can remove its contribution by a simple projection $M\tilde {y} = Q(M\tilde {\Gamma }) My$. However, since the mask destroys the orthogonality between the dictionary and the low-rank component, we do not get only the sparse contribution *M* *Φ*_*I*_ *x*(*I*) but also a (small) contribution of the low-rank component, $Q(M\tilde {\Gamma }) M\Phi _{I} x(I) = M\Phi _{I} x(I) - P(M\tilde {\Gamma }) M\Phi _{I} x(I)$. Thus, to stably estimate which part of an atom in the support has not been captured yet, we need to remove also the low-rank contribution and in our update rule replace the projection onto the current estimate of the corrupted atoms in support with the projection onto these and the (estimated) corrupted low-rank component, $P\left (M_{n}{\Psi }_{I_{n}^{t}}\right) \rightarrow P\left (M_{n}(\tilde {\Gamma },{\Psi }_{I_{n}^{t}})\right)$. Further, to ensure that the output dictionary is again orthogonal to the low-rank component, we project the updated atoms onto the orthogonal complement of the (estimated) low-rank component. Putting it all together, we arrive at the following modified algorithm.



Before we can start testing the modified algorithm, we still need to develop a method for actual recovery of the low-rank component from the corrupted data, which is presented in the next section.

## Recovery of the low-rank component

As already mentioned, in the case of uncorrupted signals, the low-rank component can be straightforwardly removed, since *Γ* will correspond to the *L* left singular vectors associated to the largest *L* singular values of the data matrix. In the case of corrupted signals, this is no longer possible since the action of the corruption will distort the left singular vectors in the direction of the more frequently observed coordinates. To counter this effect, one would have to include the mask information in the singular value decomposition. This is, for instance, done by Robust PCA which was developed for the related problem of low-rank matrix completion [33]. Unfortunately, one of the main assumptions therein is that the corruption is homogeneously spread among the coordinates, which might not be the case in our setup. To recover the low-rank component, we will, therefore, pursue a different strategy.

Let us assume for a moment that we are looking for only one low-rank atom, *L*=1. One interpretation of all (masked) signals having a good part of their energy captured by the (masked) low-rank atom is to say that all (masked) signals are 1-sparse in a dictionary of one (masked) atom. Since we already have an algorithm to learn dictionaries from corrupted signals, we can also employ it to learn the low-rank atom. Moreover, since we have an algorithm to learn dictionaries from corrupted signals that contain a low-rank component, we can iteratively learn the low-rank component atom by atom. Adapting the algorithm also leads to some simplifications. After all, we do not need to find the sparse support, since (almost) all signals are expected to contain the one new atom. Summarising these considerations, we arrive at the following algorithm.



Note that for the first low-rank atom in each iteration, the update rule reduces to a summation of the signals aligned according to $\text {sign}\left (\left \langle {\hat \gamma _{\ell }},{M_{n} y_{n}}\right \rangle \right)$. Under the assumption that the size of the low-rank component is much smaller than the sparsity level, the proposed iterative approach provides a simple tool for the low-rank component reconstruction, which is stable under non-homogenous corruption of the data. After having presented both algorithms, we will turn to testing our algorithms on synthetic and image data.

## Results

### Numerical simulations on synthetic data

In this section, we present two types of experiments on synthetic data. In the first experiment, we test the performance of the adapted version of the algorithms compared to their original counterparts. In the second experiment, we explore the connection between spikiness of the dictionary and recoverability by ITKrM(M).

#### Gains of incorporating mask information

We first compare the performance of the adapted algorithms to their original counterparts on synthetic signals. The original counterpart, which does not use mask information, performs singular value decomposition for low-rank recovery and uses ITKrM for dictionary learning. We look at two representation pairs, consisting of a low-rank component and a dictionary, and test the recovery using 6-sparse signals with corruptions of two types, random erasures and burst errors.

**Dictionary and low-rank component:** The first representation pair corresponds to the discrete cosine transform (DCT) basis in ${\mathbb {R}}^{d}$ for *d*=256. As low-rank component, we choose the first two DCT atoms, that is the constant atom and the atom corresponding to an equidistant sampling of the cosine on the interval [0,*π*), while the remaining basis elements form the dictionary. For the second pair, we construct the low-rank component by choosing two vectors uniformly at random on the sphere in ${\mathbb {R}}^{d}$ for *d*=256 and setting *Γ* the closest orthonormal basis as given by the singular value decomposition. To create the dictionary, we then choose another 1.5*d* random vectors uniformly on the sphere, project them onto the orthogonal complement of the span of *Γ* and renormalise them. These two representation pairs exhibit different complexities. The first forms an orthonormal basis, thus is maximally incoherent, and every element has $\|\gamma _{\ell }\|_{\infty } = \|\phi _{k}\|_{\infty } = \sqrt {2/d}\approx 0.088$. The second dictionary is overcomplete with coherence 0.2788 and the supremum norm of both the low-rank and the dictionary atoms varies between 0.1529 and 0.2754 and averages at 0.1897.

**Signals:** To create our signals, we use the signal model in (2) with a particular choice of distributions for the sparse and low-rank coefficients, the scaling factor and the noise, described in Table 1. For the first experiment, we set the parameters to *e*_*Γ*_=1/3, *b*_*Γ*_=0.15, *S*=6, *b*_*S*_=0.1, ${\rho }=1/(4\sqrt {d})$ and *s*_*m*_=4, resulting in 6-sparse signals with dynamic coefficient range between 1 and 0.9^−6^≈1.88 and the low-rank component containing a third of the energy. The signal-to-noise ratio is 16, and the scaling is uniformly distributed on [0,4].

**Table 1:** Signal model

**Signal model**		
Given the generating low-rank component *Γ* and dictionary *Φ*, our signal model further depends on six coefficient parameters,		
*e*_*Γ*_	-	the energy of the low-rank coefficients,
*b*_*Γ*_	-	defining the decay factor of the low-rank coefficients,
*S*	-	the sparsity level,
*b*_*S*_	-	defining the decay factor of the sparse coefficients,
*ρ*	-	the noise level and
*s*_*m*_	-	the maximal signal scale.
Given these parameters, we choose a low-rank decay factor *c*_*Γ*_ uniformly at random in the interval [1−*b*_*Γ*_,1]. We set ${v}(\ell) =\sigma _{\ell } c_{\Gamma }^{\ell }$ for 1≤*ℓ*≤*L*, where *σ*_*ℓ*_ are iid uniform ± 1 Bernoulli variables, and renormalise the sequence to have norm ∥*v*∥_2_=*e*_*Γ*_. Similarly, we choose a decay factor *c*_*S*_ for the sparse coefficients uniformly at random in the interval [1−*b*_*S*_,1]. We set $x(k) = \sigma _{k} c_{S}^{k}$ for 1≤*k*≤*S*, where *σ*_*ℓ*_ are iid uniform ± 1 Bernoulli variables, and renormalise the sequence to have norm ∥*x*∥_2_=1−*e*_*Γ*_. Finally, we choose a support set *I*={*i*_1_…*i*_*S*_} uniformly at random as well as a scaling factor *s* uniformly at random from the interval [0,*s*_*m*_] and according to our signal model in (2) set		
$y= {s} \cdot \frac {\Gamma {v} + \Phi _{I} x +{r}}{\sqrt {1+\|{r} \|_{2}^{2}}},$		
where *r* is a Gaussian noise vector with variance *ρ* ^2^ if *ρ*>0.		

**Corruption:** We consider two types of corruptions, whose distributions are described in Table 2. The random erasure patterns depend on four parameters determining (the difference in) the erasure probabilities of the first and second half of the coordinates (*p*_1_,*p*_2_) and one half and the other half of the signals (*q*_1_,*q*_2_). The expected average corruption corresponds to $1-{\mathbb {E}}\left (\sum _{k} M(k,k)\right) = 1- (p_{1}+p_{2})(q_{1}+q_{2})/4$ and in our experiments varies between 10 and 90%.

**Table 2:** Mask models

**Erasure model**		
Our erasure model depends on four parameters,		
*p*_1_	-	the relative signal corruption of the first half of coordinates,
*p*_2_	-	the relative signal corruption of the second half of coordinates,
*q*_1_	-	the corruption factor of one half of the signals and
*q*_2_	-	the corruption factor of the other half of the signals.
Based on these parameters, we generate a random erasure mask as follows. First, we choose *q*∈{*q*_1_,*q*_2_} uniformly at random and determine for every entry the probability of being non-zero as *η*_*j*_=*q* *p*_1_ for *j*≤*d*/2 and *η*_*j*_=*q* *p*_2_ for *j*>*d*/2. We then generate a mask as a realisation of the independent Bernoulli variables *M*(*j*,*j*)∼*B*(*η*_*j*_), that is *P*(*M*(*j*,*j*)=1)=*η*_*j*_.		
**Burst error model**		
Our burst error model depends on four parameters,		
*p*_*T*_	-	the probability of a burst of length *T*,
*p*_2*T*_	-	the probability of a burst of length 2*T*,
*T*	-	the burst length and
*q*	-	the probability of the burst starting in the first half of the coordinates.
Based on these parameters, we generate a burst error mask as follows. First, we choose a burstlength *τ*∈{0,*T*,2*T*} according to the probability distribution prescribed by {*p*_0_,*p*_*T*_,*p*_2*T*_}, where *p*_0_=1−*p*_*T*_−*p*_2*T*_. We then decide according to the probability *q* whether the burst start *t* occurs among the first half of coordinates, *t*≤*d*/2, or the second half, *t*>*d*/2. Finally, we draw the burst start *t* uniformly at random from the chosen half of coordinates and in a cyclic fashion set *M*(*j*,*j*)=0 whenever *t*≤*j*<*t*+*τ* or *j*<*t*+*τ*−*d* and *M*(*j*,*j*)=1 else.		

The burst error patterns also depend on four parameters determining the burstlength *T*, the probability of no burst and a burst of size *T* or of size 2*T* occurring (*p*_0_,*p*_*T*_,*p*_2*T*_ where *p*_0_=1−*p*_*T*_−*p*_2*T*_), as well as the probability of the burst occurring among the first half of the coordinates (*q*). In our experiments, we consider burstlengths *T*=64,96 with varying burst location and occurrence probabilities, leading to an empirical average corruption varying between 10 and 60%.

**Experimental setup:** We first learn the low-rank component and then the dictionary always using random initialisations. In particular, to learn the low-rank component with the adapted algorithm, we use 10 iterations for every atom and 30,000 (new) signals per iteration. As initialisation, we use a vector drawn uniformly at random from the sphere in the orthogonal complement of the low-rank component recovered so far. For the unadapted low-rank recovery, we use a singular value decomposition, where the low-rank component corresponds to the first *L* left singular vectors of the 30,000 signals generated for the adapted algorithm. As measure for the final recovery error, we use the operator norm of the difference between the generating low-rank component *Γ* and its projection onto the recovered component $\tilde {\Gamma }$, that is $\|\Gamma - P(\tilde {\Gamma }) \Gamma \|_{2,2}$. This corresponds to the worst-case approximation error of a signal in the span of the generating low-rank component by the recovered one.

We then learn the dictionary using 100 iterations of ITKrM(M) and 100,000 (new) signals per iteration from a random initialisation, where the initial atoms are drawn uniformly at random from the sphere in the orthogonal complement of the respective low-rank component. We measure the recovery success by the percentage of recovered or rather not recovered atoms, where we use the convention that a generating atom *ϕ*_*k*_ is recovered if there exists an atom $\tilde {\psi }_{j}$ in the output dictionary $\tilde {\Psi }$ for which $|\left \langle {\phi _{k}},{\tilde {\psi }_{j}}\right \rangle |\geq t$ for *t*=0.99.

**Fig. 2:**
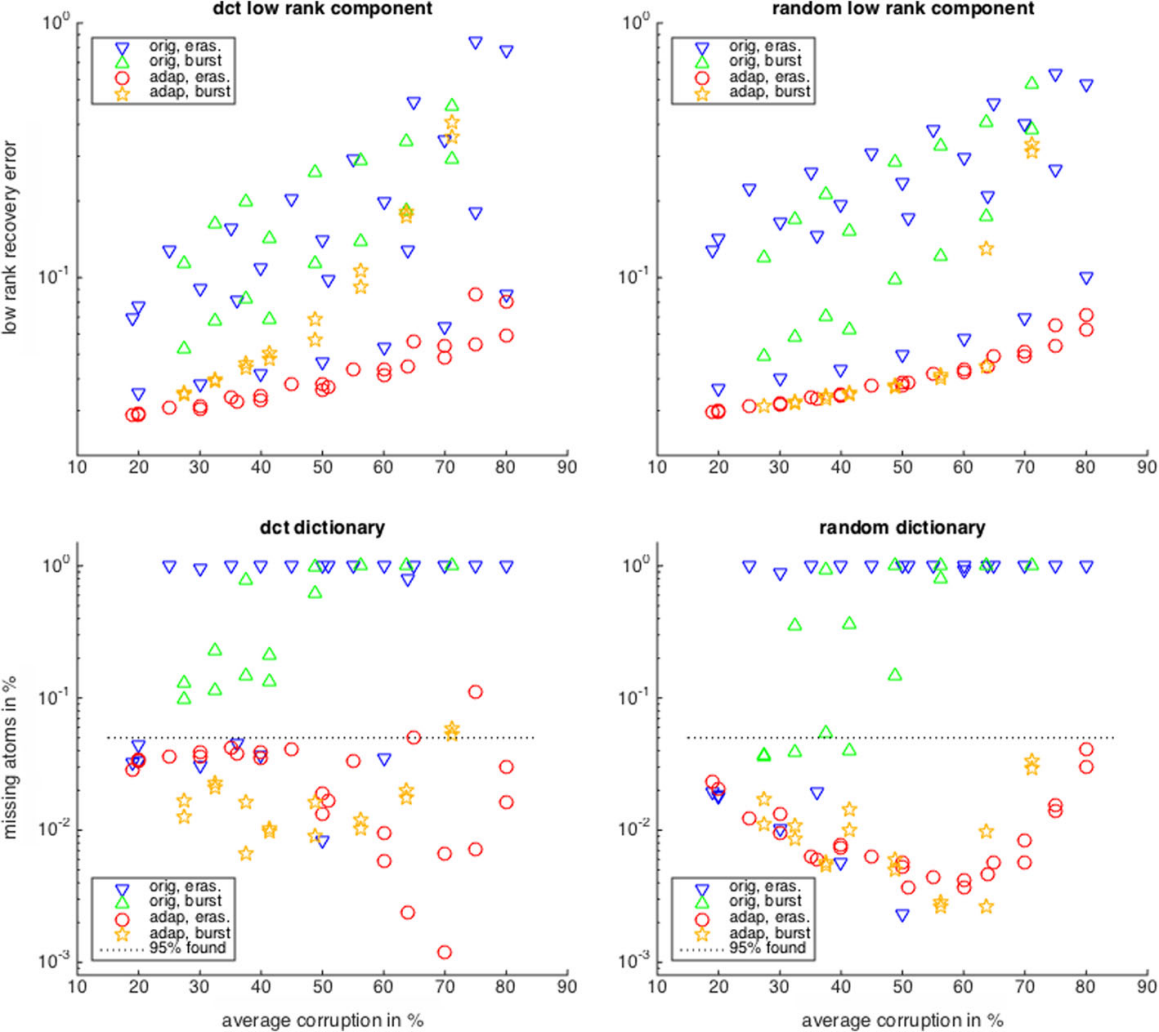
Recovery performance of the corruption adapted versus the unadapted learning algorithms for the DCT (left) and the random (right) representation pair. The recovery performance is measured in terms of the low-rank recovery error (top) and the percentage of unrecovered dictionary atoms from a random initialisation (bottom)

Figure 2 shows the recovery results for various corruption levels using the corruption-adapted algorithms (ITKrMM) and their unadapted counterparts (ITKrM). We can see that for both representation pairs, incorporating the corruption information into the learning algorithms clearly improves the performance. Another fact immediately visible is that for the adapted algorithms, the success rates differ for the two erasure modalities and decrease with increasing corruption level. However, the success rates do not depend much on the particular distribution of the erasures or bursts as long as they lead to the same average corruption level. In contrast, the success rates of the unmodified algorithms depend very much on the corruption distribution, and signals with similar average corruption can lead to very different error rates.

We also observe that corruption can improve the recovery rates of both the unmodified and the modified algorithms. A similar phenomenon has already been observed for ITKrM in connection with noise and a lower sparsity level [29]. While one might expect the global recovery rates to decrease with increasing noise and increasing *S*, they actually increase. The reason for this is that a little bit of noise or lower sparsity, like a little bit of corruption, breaks symmetries and suppresses the following phenomenon. Two atoms converge to the same generating atom, and therefore, another atom has to do the job (is a 1:1 linear combination) of two generating atoms. For uncorrupted signals, there are ongoing efforts to alleviate this phenomenon with replacement strategies, which will have a straightforward extension to corrupted signals.

To find out when we gain most from incorporating the mask information, let us have a more detailed look at the recovery rates for different types of parameter settings. Among the random erasures, we distinguish 4 types. ‘type00’ indicates that *p*_1_=*p*_2_ with *p*_1_ varying between 0.2 and 0.8 and *q*_1_=*q*_2_=1, leading to a uniform erasure probability for all coordinates and all signals. ‘type20(30)’ indicate that *p*_2_=*p*_1_+0.2(0.3) with *p*_1_ varying between 0.1 and 0.7(0.6) and again *q*_*i*_=1, leading to higher erasure probabilities for the first half of the coordinates, which are however uniform across signals. Finally, ‘type22’ indicates that *p*_2_=*p*_1_+0.2 and *q*_*i*_=*p*_*i*_ for *p*_1_ varying between 0.4 and 0.8, leading to different erasure probabilities across coordinates and across signals.

Among the burst errors, we distinguish between ‘type5’ corresponding to a uniform burst distribution and ‘type7’ corresponding to a 0.7 probability of the burst occurring in the first half of the coordinates. For each type, we consider the burstlength *T*=64 with probabilities (*p*_*T*_,*p*_2*T*_)∈{(0.5,0.3),(0.7,0.3),(0.5,0.5)} leading to corruptions between 20 and 40% and the burstlength *T*=96 with the same pairs and additionally (*p*_0_,*p*_*T*_)∈{(0.3,0.7),(0.1,0.9)} leading to corruptions between 40 and 75%.

**Fig. 3:**
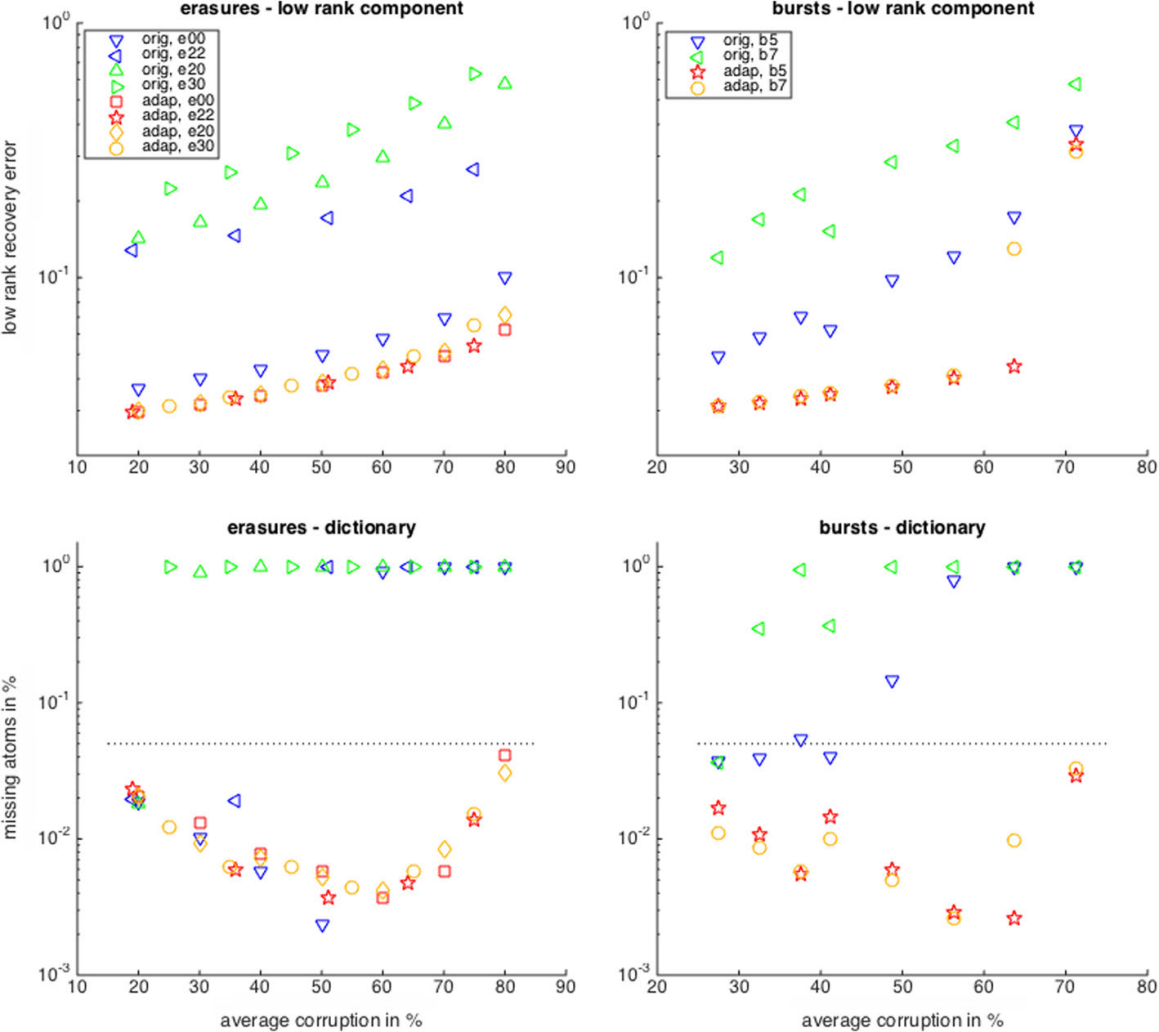
Detailed recovery performance of the corruption adapted versus the unadapted learning algorithms for the random low-rank component (top) and dictionary (bottom) for random erasures (left) and burst errors (right)

For conciseness, we focus on the random low-rank component and dictionary (Fig. 3). Distinguishing between the different types, we can now see that incorporating the corruption information gives the highest benefits when the corruption is most unevenly distributed over the signal coordinates. So, for the evenly distributed random erasures and burst errors, ‘type00’ and ‘type5’, the low-rank component is still recovered by both the unadapted and the adapted algorithm, but as soon as there is intercoordinate variance in the corruption level, type20/22/30’ and ‘type7’, the unadapted algorithm starts to lag behind. For the dictionary recovery, the unadapted algorithm only does well for homogeneous corruption, ‘type00’ and ‘type5’, until about 50% corruptions but breaks down for higher corruption levels or for intercoordinate variance of the corruption, ‘type20/22/30’ and ‘type7’.

#### Spikiness and recoverability

The second experiment explores the sensitivity of the adapted algorithms to the flatness/spikiness of the representation pairs, measured by ∥*γ*_*ℓ*_∥_*∞*_ and ∥*ϕ*_*k*_∥_*∞*_. This is done by looking at the recovery of representation pairs, which form orthonormal bases and whose atoms have their energy concentrated on supports of size *m* for *m*=4,8,16,32,64,128,256.

**Dictionaries and low-rank components:** For a given support size *m*, we choose *d* vectors *z*_*k*_ from the unit sphere in ${\mathbb {R}}^{m}$ and *d* supports *I*_*k*_=*i*_1_…*i*_*m*_ of size *m* uniformly at random and set *B*(*I*_*k*_,*k*)=*z*_*k*_ and zero else. We then calculate the closest orthonormal basis to *B* using the singular value decomposition. The first two elements of this orthonormal basis are chosen as the low-rank component, while the remaining elements form the dictionary.

**Fig. 4:**
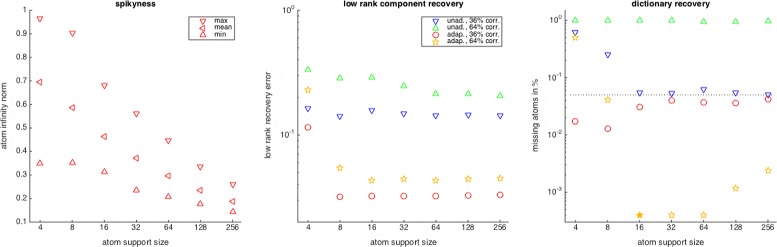
Atom spikiness (left) as well as recovery of the random low-rank component (middle) and random dictionary (right) of the corruption adapted versus the unadapted learning algorithms with varying atom support sizes. Two types of random erasure patterns leading to 36 and 64% corruption are used

**Signals, corruptions and setup:** For the signal generation, we use the same parameters as in the last experiment, and for the corruption, we use the random erasure masks of ‘type22’ with *p*_1_=*q*_1_=0.5/0.7 and *p*_2_=*q*_2_=0.7/0.9 corresponding to 36 and 64% of corruption. The experimental setup for the recovery of each representation pair is again as in the previous experiment. Figure 4 shows the spikiness of the representation pairs for various support sizes as well as the corresponding recovery results for the two corruption types. Let us first point out that our construction based on decreasing atom support sizes indeed leads to representation pairs with increased spikiness. As usual, the recovery errors incurred by the modified algorithms are much lower than those of the unmodified ones. For the low-rank component, the recovery error is very stable and only starts to deteriorate for *m*=4, when the low-rank atom carrying less energy is indeed almost a spike, ∥*γ*_2_∥_*∞*_=0.8997, meaning 80% of its energy are concentrated on one coordinate. Also, for the dictionary recovery, the robustness to spikiness of the adapted algorithms is quite surprising. So, for the low corruption level (36%), we always recover more than 95% of the dictionary atoms, and for the higher corruption level (64%), recovery only fails for *m*=4. As in the previous experiment, we observe the effect that spikiness like corruption can lead to better global recovery rates. The effect is more pronounced for the higher corruption level (64%), where for *m*=16, we even have 100% recovery.

Before turning to experiments on image data, let us mention that we also briefly investigated the effect of the signal scaling on the recovery rates of the modified algorithms for the DCT representation pair and the ‘type22’ erasure mask with 36% corruption, with the same setup as in the first experiment, but found that there was no strong influence. That is, for *s*_*m*_ varying between 2 and 128, the low-rank recovery error varies between 0.031 and 0.036 and the atom recovery rates stay between 95 and 96%.

Similarly, exploring the effect of the sparsity level *S*, we do not gain much more insights over the experiments already conducted in the uncorrupted case [29]. So, fixing all mask and signal parameters except for the sparsity parameter *S*, which increases from 4 to 16, the low-rank recovery error stays constant while the number of recovered dictionary atoms increases.

In order not to overload the paper, we do not detail these experiments here but refer the interested reader to the ITKrMM MATLAB toolbox^1^, which can be used to reproduce all the presented experiments and many more.

### Numerical simulations on image data

In this section, we will learn dictionaries on image data, more precisely on image patches, and compare the learned dictionaries to those learned by wKSVD and BPFA as well as to analytic dictionaries. The first subsection consists of a comparison of the learned dictionaries and low-rank components in terms of coherence, supremum norm, sparse approximation qualities and the computational cost of the algorithms, while in the second subsection, we will use them for inpainting, meaning the reconstruction of the missing part in an image.

#### Dictionaries for image data

In the first experiment, we compare the ITKrMM dictionaries to those learned with wKSVD and BPFA. Weighted KSVD [30, 31] is an adaption of the original KSVD algorithm [9], intended to refine a prelearned dictionary based on available corrupted data that can be then used for inpainting, which we will discuss in more details in the next subsection. Similarly, BPFA [32], which is a nonparametric Bayesian method, can be used to learn dictionaries both from corrupted and uncorrupted data, where in the case of corrupted data, the dictionary is used for inpainting.

**Data:** For our experiments, we consider the grayscale images *Barbara* and *Peppers* of size 256×256, which we corrupt by erasing each pixel independently with probability 0.3 or 0.5 resulting in 30 resp. 50% erased pixels on average. We then extract all available 8×8 patches from the corrupted image as well as the corresponding mask and give the vectorised corrupted patch/mask pairs to the learning algorithms.

**Algorithmic setup:** Via ITKrMM, we first learn the low-rank component of size *L*=1,3,7, and a dictionary of size *K*=2*d*−*L*, resulting in a system with redundancy 2. We set the sparsity level in the dictionary learning to *S*=8−*L* for *L*=1,3 corresponding to an overall sparsity *L*+*S*=8 and to *S*=5 for *L*=7, corresponding to an overall sparsity *L*+*S*=12. For wKSVD, we use the setup corresponding to ITKrMM with *L*=1 and learn a dictionary of size *K*=2*d* with the option of keeping the first atom always equal to the constant atom *ϕ*_1_≡*c*. Since within wKSVD the contribution of the constant low-rank atom counts in the sparse approximation step, we use input sparsity level *S*=8. We use the same initialisation strategies as for the synthetic experiments, i.e. random vectors that are orthogonal to the low-rank component resp. low-rank atoms that have already been learned. This means that before subtracting the low-rank component, the initial dictionaries for ITKrMM and wKSVD are the same. For learning a low-rank atom, we use 10 iterations on all available patch/mask pairs, whereas for the dictionary learning step, we use 40 iterations on all available patch/mask pairs for both algorithms. For BPFA, we use the out-of-the-box version provided on the authors’ website to learn 128 atoms from corrupted data using 150 iterations either with the recommended initialisation based on SVD or a random one. Since BPFA is a Bayesian method, it has the advantage that no sparsity level has to be defined. Note also that the SVD initialisation makes sense in this context since due to the patch structure, the corruption is evenly spread over all patch coordinates.

**Comparison:** For comparison, we also learn dictionaries on the uncorrupted images. For KSVD with *L*=1 and BPFA, we use the same setup as described above. For KSVD with *L*>1 and ITKrM, we use a similar setup as in the synthetic experiments. This means that we choose as low-rank component the first *L* principal components (left singular vectors of the data matrix), project all training signals on the orthogonal complement of the low-rank component and then learn a dictionary of size *K*=2*d*−*L* with sparsity level *S*=5 for *L*=3,7 as well as *S*=7 for *L*=1 for ITKrM, on the projected signals.

**Fig. 5:**
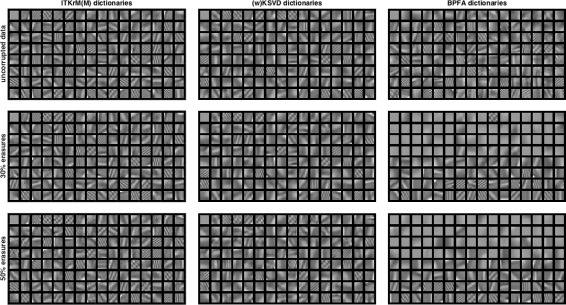
Dictionaries and low-rank atom (left upper corner) learned with ITKrM(M) (left), (w)KSVD (middle) and BPFA (right) algorithms on all 8×8 patches of *Barbara* without corruption (top), 30% erasures (middle) and 50% erasures (bottom)

**Fig. 6:**
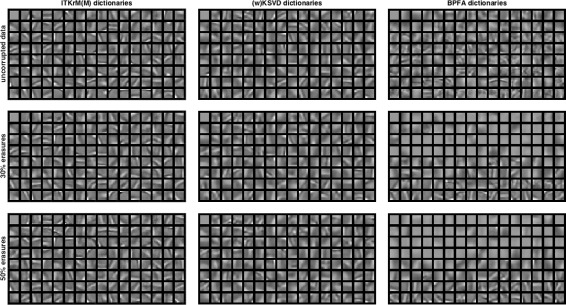
Dictionaries and low-rank atom (left upper corner) learned with ITKrM(M) (left), (w)KSVD (middle) and BPFA (right) algorithms on all 8×8 patches of *Peppers* without corruption (top), 30% erasures (middle) and 50% erasures (bottom)

**Fig. 7:**
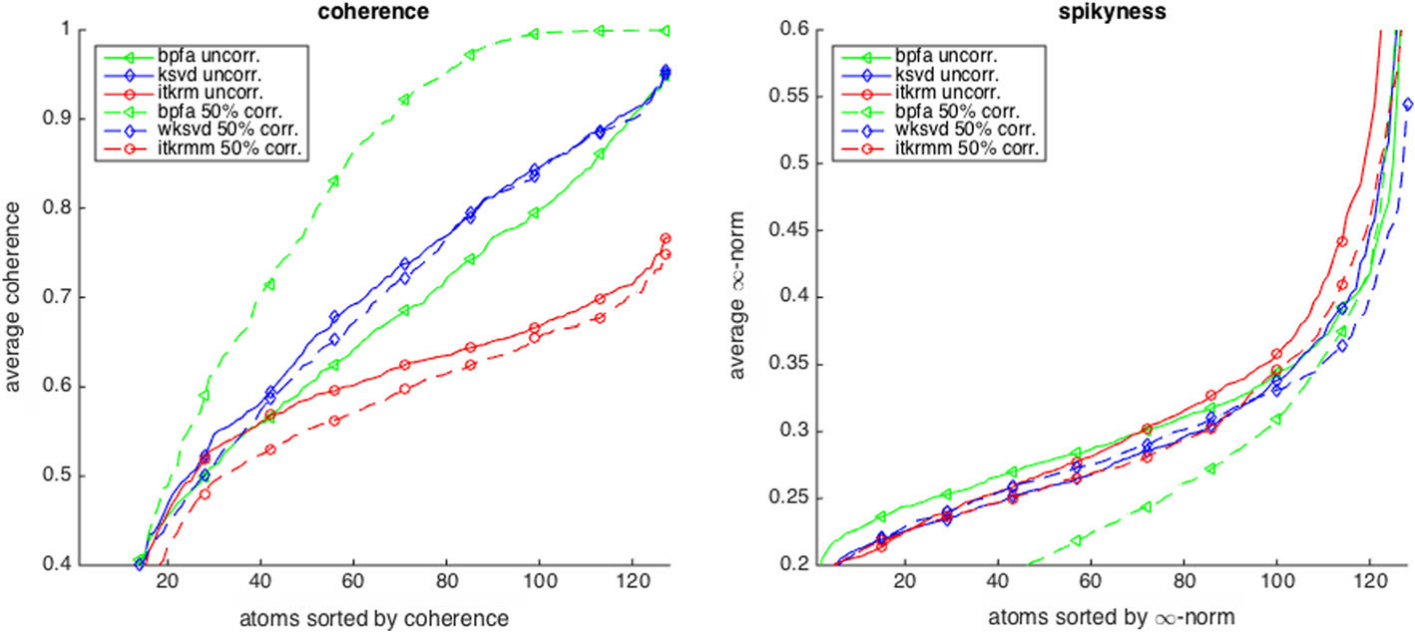
Average coherence (left) and spikiness (right) of the dictionary atoms learned on *Barbara* by BPFA, (w)KSVD and ITKrM(M) on uncorrupted data and 50% corrupted data

**Consistency:** Figures 5 and 6 show the dictionaries and if applicable low-rank components for *L*=1 learned by ITKrM(M), (w)KSVD and BPFA with SVD initialisation from uncorrupted and corrupted data. The first impression is that on uncorrupted data, the three algorithms produce quite similar dictionaries, even though ITKrM produces more high-frequency atoms than KSVD and the first BPFA atoms clearly have the structure of the principle components used in the initialisation. The next observation is that ITKrMM and wKSVD are consistent, in the sense that most of the atoms learned on corrupted data have a corresponding atom in the dictionary learned on uncorrupted data. This is not true for BPFA, where the dictionaries learned from uncorrupted and corrupted data are markedly different, the latter containing many copies of the constant atom or slight variations thereof. This is naturally reflected in the coherence and spikiness of the dictionaries. Figure 7 shows the coherence of the dictionary atoms *μ*_*k*_= max*j*≠*k*|〈*ψ*_*k*_,*ψ*_*j*_〉| and their supremum norm ∥*ψ*_*k*_∥_*∞*_ sorted and averaged over five different random mask realisation/initialisations for 0 and 50% corruption. ITKrM(M) produces the most incoherent and spikiest dictionaries, while BPFA produces the flattest dictionaries and on corrupted data also the most coherent ones. The reason for this might be that BPFA was not designed for consistency, but primarily for image processing tasks, such as inpainting, where flatness can be of advantage.

**Fig. 8:**
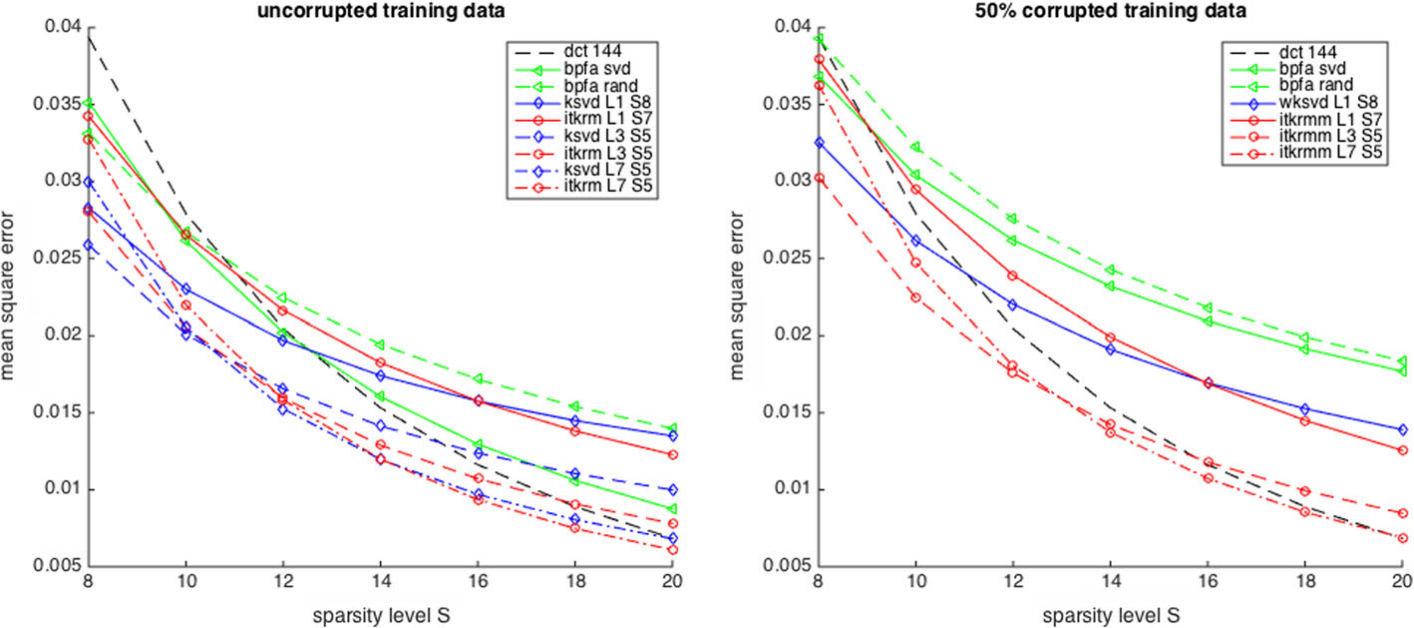
Approximation quality of dictionaries with low-rank components of various sizes on *Barbara*, DCT144 as well as BPFA, (w)KSVD and ITKrM(M) learned on uncorrupted data (left) and on 50% corrupted data (right)

**Approximation quality and low-rank components:** To illustrate the importance of integrating low-rank components into dictionary learning on real data, we test how sparsely the various representation systems learned on *Barbara* approximate all image patches of *Barbara*. For every dictionary—low-rank—component pair, containing 128 atoms, learned either on clean or corrupted data, we calculate the mean square error achieved by approximating all clean patches, using orthogonal matching pursuit (OMP) and different sparsity levels from 8 to 20. Figure 8 shows the results averaged over five different initialisations and corruption patterns where applicable. Our first observation is that the dictionaries learned by KSVD and ITKrM on clean data with *S*=5 and after removing a low-rank component of size *L*=3 or *L*=7 perform best, indicating the importance of removing the low-rank component to get a well-conditioned dictionary. Similarly, the BPFA dictionary with SVD initialisation performs much better than the randomly initialised one. We also see that the advantage of the learned dictionaries over the overcomplete DCT for small *S* gradually decreases and vanishes at *S*=20. Comparing to the dictionaries learned from corrupted data, we see that the wKSVD and ITKrMM dictionaries perform almost equally to their counterparts KSVD and ITKrM, the ITKrMM dictionaries giving the best performance, as the algorithm can also handle low-rank components with *L*>1. In contrast, the performance of the BPFA dictionaries degrades quite a lot, regardless of the initialisation. This is to be expected as the many copies of the flat atom, we have seen in Figs. 5 and 6, essentially reduce the size and with it the approximation power of the dictionary.

**Computation time:** As both ITKrMM and wKSVD produce consistent and incoherent dictionaries with good approximation properties also from corrupted data, which is the main interest of this paper, we further compare them with respect to computational cost and memory requirements. The cost per training signal of one iteration of ITKrMM consists of the inner product between the dictionary and signal, *O*(*d* *K*), the pseudo-inverse of a *d*×*S* matrix together with some matrix vector multiplications for calculating the residual, *O*(*S* ^2^ *d*+*S* *d*), and the update of *S* atoms based on the residual resp. *S* weight vectors based on the mask, *O*(*S* *d*). All in all for *N* training signals, this amounts to a computational cost of *O*(*d* *K* *N*+*S* ^2^ *d* *N*) operations per iteration.

On the other hand, the cost per iteration of wKSVD consists of sparsely approximating *N* signals with masked OMP (see Algorithm 4) and the dictionary update. The cost of OMP per signal is lower bounded by the cost of the inner products between *K* atoms and the residual for *S* iterations, *O*(*S* *d* *K*), which dominates the cost of the residual updates, *O*(*S* ^2^ *d*+*S* *d*). The update of each atom involves the calculation of the largest left singular value of a matrix *Y*_*k*_ of approximate size $d\times \frac {SN}{K}$ for several iterations. Using in turn an iterative procedure for the singular vector, we can lower bound the cost of one atom update by calculating the matrix vector products $Y_{k} \left (Y_{k}^{\star } v\right)$, *O*(*d* *S* *N*/*K*). Thus, for *N* training signals, the cost per iteration of wKSVD can be lower bounded by *O*(*S* *d* *K* *N*), meaning that ITKrMM is at least by a factor min{*S*,*K*/*S*} cheaper. Note also that contrary to KSVD, the weighted version cannot be accelerated using batch OMP [34], as every mask changes the geometry of the dictionary. Both algorithms could be further optimised noting that a masked signal is projected onto $m_{n}=\|M_{n}\|^{2}_{F}$ coordinates. This means that all sparse approximation procedures could be done in ${\mathbb {R}}^{m_{n}}$ instead of ${\mathbb {R}}^{d}$, and so setting $m=\frac {1}{N} \sum {m_{n}}$, the cost estimate for ITKrMM reduces to *O*(*m* *K* *N*+*S* ^2^ *m* *N*) and for wKSVD to *O*(*S* *m* *K* *N*). In our implementations, we refrain from this option, since we doubt that in MATLAB, the multiplications by zero in full space are costlier than locating and accessing the correct coordinates.

Further comparing the memory requirements of the two algorithms, we see that ITKrMM needs about twice the size of the dictionary matrix *O*(*d* *K*). The memory requirements for wKSVD are much larger and correspond to the entire matrix of training signals, *O*(*d* *N*) or *O*(*m* *N*), since the iteratively weighted dictionary update repeatedly accesses residuals, coefficients and masks. This also means that wKSVD cannot be used sequentially like ITKrMM.

This significant reduction in computational cost and memory requirements represents the main advantage of ITKrMM over wKSVD. In order to exemplify it, we present in Table 3 the average speed-up of ITKrMM over wKSVD for *Barbara* and *Peppers* on corrupted data as well as the speed-up of the original ITKrM over KSVD, as available on the authors’ homepage. The results are averaged over 5 runs, using the setup described above. All calculations were carried out in single thread mode on the UIBK LEO3 computing cluster consisting of 1944 Intel Xeon (Gulftown) computing cores each equipped with 24GB RAM. For completeness, we also include a comparison to BPFA. We see that both on uncorrupted and corrupted data, ITKrMM is about 11 times faster than wKSVD, i.e. wKSVD takes about 3.5 h, while ITKrMM takes only about 18 min to learn a dictionary.

**Table 3:** Speed-up of ITKrM(M) over (w)KSVD and BPFA, corresponding to the average runtime of wKSVD/BPFA divided to that of ITKrMM using all available (corrupted) image patches of Barbara and Peppers

Corr. (%)	BPFA		(w)KSVD	
	Barb.	Pepp.	Barb.	Pepp.
0	1.75	1.94	10.61	11.46
30	1.02	1.20	11.07	11.50
50	1.53	1.92	11.30	12.35

#### Inpainting

To demonstrate the practical value of the ITKrMM algorithm, we here conduct an image inpainting experiment. Inpainting is the process of filling in missing information or holes in damaged signals, and our motivating task, the prediction of blood glucose levels, can be cast as inpainting problem. Image inpainting, in particular, is used for restoration of old analogue paintings, denoising of digital photos, and for removal of objects like text or date stamps from images and has become an active field of research in the mathematical and engineering communities, with a variety of specifically developed methods and approaches [35]. Most of the existing approaches for inpainting are based on either variational approaches pioneered by Sapiro [36] or exploit image statistical and self-similarity priors as introduced by Efros [37]. With the advent of sparse representations and compressed sensing, sparsity-based inpainting has gained popularity in the recent years.

Since the primary goal of this paper is to evaluate the ITKrMM algorithm as a consistent and computationally efficient method for dictionary learning from incomplete data, we perform a thorough comparison of the ITKrMM-based inpainting algorithm with other sparsity-based inpainting methods. In particular, we compare to the inpainting schemes based on wKSVD and BPFA dictionaries as well as analytic dictionaries such as the DCT basis and the overcomplete DCT frame with 144 atoms. In all cases, we show that our results are mostly better than the ones of BPFA and wKSVD, with a large reduction of the computational costs with respect to the latter. We also show that ITKrMM-based inpainting leads to better results compared to the ones obtained with the DCT dictionaries or more advanced methods, also based on analytic dictionaries, such as morphological component analysis (MCA)[6]. Last, we briefly compare our results to PLE [38], a state-of-the-art inpainting method for natural images. PLE is based both on structured sparsity and statistical priors on the sparse coefficient distribution and is known to outperform all simple sparsity based schemes.

**Sparsity-based inpainting:** Sparsity-based inpainting relies on the concept that the signal *y* is *S*-sparse in a dictionary *Φ*, and therefore, the damaged signal *My* is sparse in the damaged dictionary *M* *Φ*, that is for |*I*|≤*S*

5 $$\begin{array}{*{20}l} y \approx \Phi_{I} x_{I} \quad \Rightarrow \quad My \approx M\Phi_{I} x_{I}. \end{array} $$

To reconstruct the original signal one therefore simply needs to recover coefficients $\tilde {x}_{I} \approx x_{I}$ by sparsely approximating *My* in *M* *Φ* and to set $\tilde {y} = \Phi \tilde {x}_{I}$. However, for the sparse approximation of *My* to recover the correct support *I*, we do not only need that the signal is very sparse *S*≪*d* but also that damaged dictionary *M* *Φ* remains incoherent, which translates to the original atoms having small supremum norm, ∥*ϕ*∥_*∞*_≪1. In summary, the sparser the representation provided and the flatter the atoms, the better the dictionary is suited for inpainting. This means that BPFA dictionaries, which have very flat atoms, as discussed in Section 5.2.1, might be better suited for inpainting than the wKSVD or ITKrMM dictionaries, which have comparatively spiky atoms, despite the fact that the latter provide sparser representations.

For sparse approximation of the coefficients, we use a slightly modified version of the well-known greedy algorithm, OMP [39, 40], which takes into account masked data. In particular, as the damaged dictionary is not normalised, we need to account for this in the OMP selection step and rescale by 1/∥*M* *ϕ*_*k*_∥_2_, similar to thresholding in the ITKrMM algorithm. Without this renormalisation, less damaged atoms take precedence over better fitting ones. The algorithm to which we refer as mOMP is described in Algorithm 4.



The inpainted image is obtained by first reconstructing every damaged image patch via mOMP and then reconstructing the complete image by averaging every pixel over all reconstructed patches in which it is contained.

**Images:** We consider six grayscale images, *Barbara*, *Peppers*, *House*, *Cameraman*, *Mandrill* and *Pirate*, of size 256×256. The images are corrupted by erasing each pixel iid with probability 0.3,0.5 or 0.7, resulting in 30, 50 or 70% erased pixels on average.

**Learning setup**: The dictionary learning setup is the same as in the experiments for 30 and 50% corruption levels in Section 5.2.1, where for ITKrMM, we consider low-rank components of size *L*=1 and *L*=3, abbreviated as ITKrMM1 and ITKrMM3 respectively. For 70% corruption, we reduce the sparsity level of ITKrMM and wKSVD in the learning stage to *S*=3 and *S*=4, respectively, and use only *L*=1. This reduction is necessary because sparse approximation becomes difficult if the dictionary is coherent $\mu \gtrsim 1/S$. In effective dimension (average number of uncorrupted pixels per signal) 64·0.3≈19, a perfectly incoherent dictionary with 128 atoms already has coherence of at least 0.19>1/8, due to the Welch bound $\mu \geq \sqrt {\frac {K-d}{d(K-1)}}$. A randomly erased dictionary adapted to the data will be even more coherent, which renders learning with *S*=7/8 risky.

**Inpainting sparsity level**: We perform sparsity-based inpainting using mOMP with sparsity levels 4:4:24 and dictionaries learned by ITKrMM and wKSVD, the DCT basis, as well as an overcomplete DCT frame with 144 atoms. For BPFA, we report the results of both the accompanying inpainting procedure as provided in [32], as well as the mOMP-based scheme used for the other dictionaries, abbreviated as BPFAomp. In the case of 70% erasures, we also include results of sparsity-based inpainting with a slight twist to deal with spikiness of the atoms. In particular, to prevent inpainting with unreliable, ill-preserved atoms, we modify the mOMP selection step, so for $m = \|M\|_{F}^{2},$ we find

$$\begin{array}{*{20}l} \max_{k} \frac{|\left\langle{r},{M\phi_{k}}\right\rangle|}{\|M\phi_{k}\|_{2}} \quad \text{over} \quad k:\:\: \|M\phi_{k}\|_{2}\geq \frac{m}{d} \|\phi_{k}\|_{2}. \end{array} $$

The results in Tables 4 and 5 achieved with this modification are marked with an asterisk (*), for example ITKrMM*. We further compare the methods to MCA [6] as it is based on sparsity in a dictionary made of two analytical orthonormal bases, such as wavelets, curvelets and DCT, for instance. Specifically, after comparing the performance of different combinations of bases for MCA, we present only the best results achieved by the undecimated discrete wavelet transform and curvelets. This combination has also been used by the authors for one of the inpainting examples in the original code.

**Table 4:** Comparison of the PSNR (in dB) for inpainting of images with various corruption levels based on analytic dictionaries, DCT, MCA, and dictionaries learned on all available corrupted image patches, BPFA, BPFAomp, wKSVD, and ITKrMM (modified inpainting is marked with a *)

	Algorithm	Bar.	Cam.	Hou.	Man.	Pepp.	Pir.
30% corruption	Noisy Im.	11.17	10.81	10.11	10.82	11.18	11.70
	DCT64	*37.49*	32.66	41.89	30.60	39.12	35.40
	DCT144	37.08	32.41	41.49	30.86	38.90	35.42
	MCA	35.89	32.45	39.62	28.38	35.59	33.35
	BPFA	34.76	32.08	39.76	29.58	37.92	34.38
	BPFAomp	35.36	32.23	41.09	30.81	38.66	35.42
	wKSVD	35.87	32.62	41.42	30.41	38.64	35.09
	ITKrMM1	36.12	32.80	41.97	30.85	39.20	35.60
	ITKrMM3	37.16	**3** **3** **.** **0** **4**	**4** **2** **.** **3** **0**	**3** **0** **.** **9** **2**	**3** **9** **.** **8** **0**	**3** **6** **.** **0** **8**
50% corruption	Noisy Im.	8.95	8.59	7.88	8.60	8.96	9.47
	DCT64	32.72	28.56	36.65	26.99	34.01	31.10
	DCT144	32.46	28.46	36.40	27.25	33.93	31.17
	MCA	32.50	28.99	36.54	25.34	32.35	29.86
	BPFA	32.97	28.89	37.71	27.25	35.29	31.89
	BPFAomp	32.98	28.87	37.88	27.29	35.41	32.18
	wKSVD	33.23	**2** **9** **.** **5** **5**	**3** **8** **.** **2** **1**	27.79	**3** **5** **.** **4** **1**	32.12
	ITKrMM1	33.28	29.44	37.75	27.96	35.31	32.14
	ITKrMM3	**3** **3** **.** **8** **2**	29.48	38.04	**2** **7** **.** **9** **7**	35.30	**3** **2** **.** **2** **6**
70% corruption	Noisy Im.	7.48	7.13	6.42	7.13	7.50	8.01
	DCT64	28.21	24.86	31.49	24.29	29.05	27.21
	DCT144	28.09	24.81	31.37	24.44	28.81	27.32
	MCA	28.74	25.71	33.42	23.29	28.56	26.55
	BPFA	29.40	25.74	33.56	24.93	**3** **1** **.** **4** **3**	28.77
	BPFAomp	29.22	25.61	33.05	25.10	31.12	28.63
	BPFAomp*	29.23	25.60	33.04	25.11	31.18	28.74
	wKSVD	29.70	25.89	33.96	25.09	31.17	28.76
	wKSVD*	29.74	26.02	**3** **4** **.** **0** **9**	25.09	31.32	**2** **8** **.** **8** **4**
	ITKrMM1	29.48	25.84	33.26	25.11	29.64	28.53
	ITKrMM1*	**2** **9** **.** **9** **3**	**2** **6** **.** **3** **4**	33.65	**2** **5** **.** **1** **2**	31.26	28.83

The results are averaged over 5 random masks and initialisations. The best result for each setting is marked in bold

**Table 5:** Comparison of the SSIM value (0.−) for inpainting of images with various corruption levels based on analytic dictionaries, DCT and MCA, and dictionaries learned on all available corrupted image patches, BPFA, BPFAomp, wKSVD and ITKrMM (modified inpainting is marked with a *)

	Algorithm	Bar.	Cam.	Hou.	Man.	Pepp.	Pir.
30% Corr.	Noisy Im.	1689	2367	0831	1604	1630	1619
	DCT64	***9822***	***9638***	9813	9373	***9859***	9691
	DCT144	9812	9629	9814	9413	9858	9698
	MCA	9695	9500	9658	9218	9654	9552
	BPFA	9438	9388	9608	8855	9710	9452
	BPFAomp	9590	9564	9771	9400	9820	9688
	wKSVD	9659	9551	9772	9341	9812	9662
	ITKrMM1	9719	9597	9819	9418	9840	9701
	ITKrMM3	9798	9612	***9823***	***9428***	9852	***9729***
50% Corr.	Noisy Im.	1002	1617	0478	0899	1015	0946
	DCT64	9503	9207	9542	8469	9647	9215
	DCT144	9486	9194	9548	8566	9645	9239
	MCA	9424	9173	9484	8408	9511	9157
	BPFA	9284	9141	9508	8187	9612	9171
	BPFAomp	9384	9222	9607	8782	9686	9377
	wKSVD	9439	9257	9607	8786	9683	9363
	ITKrMM1	9514	9281	9651	8816	9688	9374
	ITKrMM3	***9588***	***9281***	***9657***	***8825***	***9695***	***9392***
70% Corr.	Noisy Im.	0552	0981	0268	0459	0575	0515
	DCT64	8703	8411	8976	6950	9115	8224
	DCT144	8682	8389	8982	7081	9098	8275
	MCA	8807	8592	9070	7056	9152	8363
	BPFA	8783	8587	9231	7104	9366	8604
	BPFAomp	8828	8600	9235	7614	9374	8713
	BPFAomp*	8834	8602	9238	7615	9387	8740
	wKSVD	8877	8648	9286	***7616***	9380	8750
	wKSVD*	8885	8676	9290	7615	9392	***8757***
	ITKrMM1	8937	8661	9306	7582	9222	8720
	ITKrMM1*	***9002***	***8749***	***9323***	7583	***9404***	8753

The results are averaged over 5 random masks and initialisations. The best result for each setting is marked in bold

The results of wKSVD are generated with our own implementation modified from the original KSVD algorithm, as there is no MATLAB version openly available, while those of BPFA and MCA are produced by the original software and the authors’ recommended settings.

**Comparison/error:** We measure the recovery success of the schemes by the peak signal-to-noise ratio (PSNR) and the similarity index (SSIM) between the original image *Y* and the recovered version $\tilde {Y}$. For two images $Y \text {and}~\tilde {Y}$ of size *d*_1_×*d*_2_, the PSNR in dB is defined as

$$\log_{10}\left(\frac{(\max_{i,j}Y(i,j) - \min_{i,j}Y(i,j))^{2} }{\frac{1}{d_{1} d_{2}} \sum_{i,j} (Y(i,j) - \tilde{Y}(i,j))}\right). $$

The SSIM index is defined as

$$\frac{(2\mu_{\tilde{Y}} \mu_{Y} + c_{1})(2 \sigma_{\tilde{Y} Y} + c_{2})}{(\mu_{\tilde{Y}}^{2} + \mu_{Y}^{2} + c_{1})(\sigma_{\tilde{Y}}^{2} + \sigma_{Y}^{2} + c2)}, $$

where $\mu _{\tilde {Y}},\mu _{Y}, \sigma _{\tilde {Y}}, \sigma _{Y}$ and $\sigma _{\tilde {Y} Y}$ are the local means, standard deviations, and cross-covariance for images $\tilde {Y}$ and *Y*. For the similarity index, we take the default settings for *c*_1_ and *c*_2_ with maximal image value 1. The results are averaged over 5 runs, each with a different mask and in case of ITKrMM and wKSVD different initialisations, to account for the variability between different mask realisations. For all OMP-based schemes, we only report the values corresponding to the sparsity level that gives the best result on average over the 5 trials.

Table 4 provides the PSNR values generated by all algorithms on the considered images. Inpainting with the DCT dictionaries gives relatively good results, even though the data-learned dictionaries like BPFA, wKSVD and ITKrMM outperform the DCT dictionaries all but once, the exception being *Barbara* with 30% erasures, where the very flat DCT basis is quite well suited to capture the textures.

On all other images with 30% corruptions the ITKrMM dictionaries provide the best results. In case of 50 and 70% random erasures, the wKSVD and ITKrMM dictionaries tend to divide the best performance between themselves. In particular, for more textured images like *Barbara*, *Mandrill*, and *Pirate*, ITKrMM, which tends towards high-frequency atoms, has a slight advantage, while for the smooth images like *Cameraman*, *House*, and *Peppers*, wKSVD is slightly better. We also see that BPFA with the sparse inpainting scheme improves over the original BPFA inpainting procedure for 30 and 50% corruption. For 70% corruption, this trend is reversed and BPFA even takes home the win once. Another observation is that for large corruption, even the slight twist in inpainting to balance for spikyness already improves the performance both of the ITKrMM and wKSVD dictionaries. This is especially interesting in view of comparison to state-of-the-art inpainting methods designed for images, such as the PLE algorithm [38]. On top of using a learned dictionary made of two PCA bases, PLE employs the concepts of block sparsity and some weights capturing the probability of an atom being used. Using this more refined sparsity-based inpainting procedure, PLE outperforms the generic sparsity-based scheme using ITKrMM or wKSVD by about 1dB on *House* with 50 or 70% corruption and by about 3 dB on *Barbara* 50% or corruption 70%.

For a more comprehensive comparison, we also present the average SSIM values of the reconstructed images for the various schemes in Table 5. The SSIM results are in general consistent with the ones for PSNR. However, for the 30% corruption level, inpainting with the DCT basis/frame provides slightly better values, followed by ITKrMM. Moreover, one can observe that for 50% corruption, the SSIM values for the ITKrMM algorithms are slightly better than for all other algorithms—for 70% corruption, they are better 4 out of 6 times. Compared to the PSNR results for the same level of corruption, this essentially supports our previous conclusions that the tendency of ITKrMM algorithm towards high-frequency atoms allows to recover fine details, without too much oversmoothing. In contrast, the wKSVD algorithm, which sometimes has better PSNR values but worse SSIM values, leads to smoother images.

**Fig. 9:**
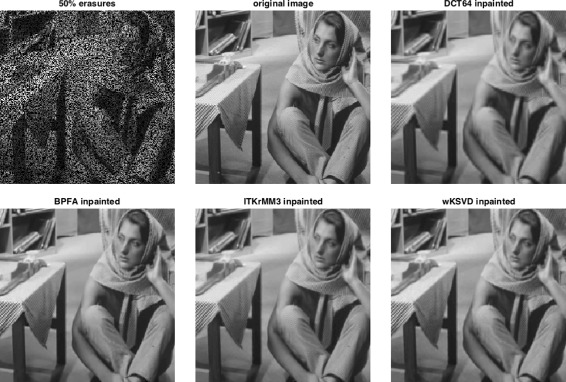
Inpainting example: Barbara. Top row, left to right: image with 50% erasures, original image and reconstruction based on the DCT basis. Bottom row, left to right, reconstruction based on dictionaries learned by BPFA, ITKrMM (*L*=3) and wKSVD

Figure 9 shows an inpainting example on *Barbara* with 50% corruption. All learned dictionaries under consideration are able to inpaint the image with similar visual quality, while inpainting with the DCT basis produces a slightly blurry image. ITKrMM generates the highest PSNR, followed by wKSVD, BPFA and DCT64, which, for instance, manifests itself in the slightly better recovery of the texture on the trousers.

## Discussion and conclusions

Inspired by real-life problems and applications, where data is incomplete and corrupted, we here extended the iterative thresholding and *K* residual means (ITKrM) algorithm for dictionary learning to learning dictionaries from incomplete/masked data (ITKrMM). To account for the presence of a low-rank component in the data, we further introduced a modified version of the ITKrMM algorithm to recover the low-rank component and adapted the ITKrMM algorithm to the potential presence of such a low-rank component. In extensive tests on synthetic data, we demonstrated that incorporating information about the corruption (missing coordinates) dramatically improves the dictionary learning performance and that ITKrMM is able to recover dictionaries from data with up to 80% corruption. We further showed that the algorithm learns meaningful dictionaries on corrupted image data and demonstrated the importance of considering the presence of a low-rank component for good approximation properties of the dictionary. We also showed that ITKrMM provides significant improvements in terms of dictionary quality and consistency compared to BPFA and in terms of computation cost/time (e.g. 18 min vs. 3.5 h) and memory requirements compared to wKSVD, a state-of-the-art algorithm for dictionary learning/refinement in the presence of erasures. Moreover, when used for inpainting, the ITKrMM dictionaries often perform better than their dictionary learning counterparts, wKSVD and BPFA, analytic dictionaries like DCT or even more advanced methods based on analytic dictionaries like MCA, leading to notable improvements for images with moderate to medium corruption level or textured images with any corruption level.

All the experiments reported in this paper can be reproduced with the freely available ITKrMM Matlab toolbox at the second author’s homepage.

One slight disappointment is that in synthetic experiments with a random initialisation, ITKrMM does not recover the full dictionary. Instead, it recovers some atoms twice, and some atoms are 1:1 linear combinations of two other ground truth atoms. This phenomenon has already been observed in the case of ITKrM, and there are ongoing efforts to counter it with replacement strategies. Research in this direction goes hand in hand with increasing the theoretical convergence radius of ITKrM derived in [29] and further opens up the road to adaptively choosing the sparsity level, the dictionary size and the size of the low-rank component. Once these strategies and the sharper analysis for ITKrM are finalised, we are planning to extend both of them to the case of corrupted data, that is ITKrMM. In particular, we want to further adapt the choice of the sparsity level and the dictionary size to the level of corruption and the amount of training data, which we expect to improve the performance of the ITKrMM algorithm for image inpainting both in terms of speed and accuracy.

Another interesting direction would be to combine ITKrMM-learned dictionaries with the inpainting scheme of PLE [38], using structured sparsity and coefficient statistics obtained in the learning. After all, one of the motivations for relying on two PCA bases rather than a dictionary seems to have been instability of dictionary learning and a lack of theoretical support.

More generally, we are interested in extending the concept of learning dictionaries from masked data to other types of corruption such as, for instance, blurring, where the resulting dictionaries can then be used for deblurring.

## Footnotes

1
https://www.uibk.ac.at/mathematik/personal/schnass/code/itkrmm.zip
